# Augmenting mesenchymal stem cell therapy for osteoarthritis via inflammatory priming: a comparative study on mesenchymal stem cells derived from various perinatal tissue sources

**DOI:** 10.3389/fcell.2023.1279574

**Published:** 2023-10-04

**Authors:** Xinzi Xia, Yue Sui, Jiawen Zhou, Shanshan Li, Xiang Ma, Jiang Jiang, Yaping Yan

**Affiliations:** ^1^ State Key Laboratory of Primate Biomedical Research, Institute of Primate Translational Medicine, Kunming University of Science and Technology, Kunming, Yunnan, China; ^2^ Department of Obstetrics, The First People’s Hospital of Yunnan Province, Kunming, Yunnan, China

**Keywords:** osteoarthritis, mesenchymal stem cells, heterogeneity, articular chondrocytes repair, IL-1β pretreatment

## Abstract

**Background:** Osteoarthritis (OA), a degenerative disease prevalent among the elderly, poses significant challenges due to its high incidence and disability rates. Regrettably, there exists a lack of effective regenerative therapies for the irreversible degradation of cartilage in OA. Mesenchymal stem cells (MSCs), known for their robust differentiation and immune regulatory capabilities, have emerged as promising candidates for OA treatment. MSCs sourced from perinatal tissues offer the dual advantage of convenience in extraction and ethical non-controversy. However, the heterogeneous nature of MSCs derived from different perinatal tissue sources gives rise to varying therapeutic indications. Moreover, the immune response of MSCs may be modulated under the influence of inflammatory factors.

**Methods:** In this study, we isolated mesenchymal stem cells from distinct parts of human perinatal tissue: umbilical cord-derived MSCs (UC-MSCs), fetal placenta-derived MSCs (FP-MSCs), and umbilical cord placental junction-derived MSCs (CPJ-MSCs). These cells were cultured *in vitro* and subjected to a 24-hour treatment with the inflammatory mediator Interleukin-1β (IL-1β). Subsequently, the MSCs were evaluated for changes in proliferation, migration, and regulatory capabilities. To assess the comparative anti-injury potential of MSCs from different sources, primary articular chondrocytes (ACs) were exposed to H_2_O_2_-induced injury and co-cultured with IL-1β-primed MSCs. Changes in the proliferation, migration, and regulatory abilities of ACs resembling those observed in OA were examined.

**Results:** Following IL-1β treatment, all three types of MSCs displayed decreased rates of proliferation and migration. Notably, their chondrogenic differentiation capacities exhibited an enhancement. Additionally, diverse MSCs exhibited a degree of efficacy in restoring damaged ACs *in vitro*. Among these, CPJ-MSCs demonstrated superior potential in promoting cartilage cell proliferation, while FP-MSCs displayed notable anti-inflammatory effects.

**Conclusion:** Our findings underscore the substantial capacity of primed FP-MSCs and CPJ-MSCs to alleviate the injury in OA-like ACs. Consequently, this study advocates for the prospective use of preconditioning strategies involving FP-MSCs and CPJ-MSCs in forthcoming OA therapies.

## 1 Introduction

Osteoarthritis (OA), a degenerative ailment predominantly affecting the elderly, poses a serious health risk, particularly in knee joint injuries (Pap and Korb-Pap, 2015). The surging aging population and the prevalence of obesity have propelled OA incidence to alarming rates. By 2020, the global count of OA patients exceeded 500 million, inflicting substantial economic burdens on individuals and healthcare systems (Sharma, 2021; Hunter et al., 2022). Clinical manifestations of OA encompass joint pain, diminished joint space, deteriorating articular cartilage, excessive bone growth, and, in advanced stages, disability (Roseti et al., 2019). The complex pathogenesis of OA implicates aging, inflammation, mechanical loads, and other factors. Post-OA onset, damaged articular cartilage experiences cumulative degeneration, accompanied by secondary synovitis, subchondral bone remodeling, and ligament and muscle contraction. Alas, the avascular, nerveless, and lymphless nature of articular cartilage compounds the challenge of restoring functional hyaline cartilage, rendering efficient regeneration elusive.

The current OA treatments primarily comprise pharmacological intervention, surgical approaches, and cell-based therapies. Pharmacotherapy seeks pain relief and inflammation mitigation through oral or intravenous agents (Anderson et al., 2014). Yet, long-term pharmacological dependence seldom promotes patient recovery and may induce side effects (Hochberg et al., 2012). Surgical strategies encompass debridement, bone marrow stimulation, and artificial joint replacement; however, their sustained therapeutic efficacy remains challenging (Gobbi et al., 2014), along with the risk of postoperative infections (Orth et al., 2012). Autologous chondrocyte implantation (ACI) has been pivotal in cell-based OA therapy (Brittberg et al., 1994), but its potential to harm donor sites and limited cell availability necessitating *in vitro* expansion underscores its limitations (Saris et al., 2014; Duan et al., 2015; Wang et al., 2018). Consequently, the quest for alternative cell sources for OA cartilage restoration prompted exploration of mesenchymal stem cells (MSCs).

MSCs, multipotent cells isolable from tissues such as bone marrow, adipose, placenta, and umbilical cord, possess robust self-renewal and differentiation capabilities (Vasanthan et al., 2020). Following injection into OA-afflicted knee joints, MSCs foster a co-culture milieu with injured chondrocytes, augmenting cartilage anabolism and extracellular matrix regulation (Najar et al., 2020). Paracrine factors secreted by MSCs, including transforming growth factor-β (TGF-β), hepatocyte growth factor (HGF), prostaglandin E2 (PGE2), platelet-derived growth factor (PDGF), and IL-10, counter inflammation and endogenous cell proliferation within the joint (Harrell et al., 2019; Zhang et al., 2021). Intra-articular MSC injection emerges as a promising OA therapy, with diverse MSC sources explored (CMatas et al., 2019; Lee et al., 2019; Soltani et al., 2019). Heterogeneity in proliferative and differentiation capacities prompts questions on indications. Compared to bone marrow-derived MSCs and adipose-derived MSCs, MSCs isolated from perinatal tissues have the advantages such as non-invasiveness and convenience of sampling. Meanwhile, studies have shown that the proliferation and differentiation ability of MSCs derived from bone marrow decrease with the increase of donor’s age (Wong et al., 2013). Perinatal tissues-derived stem cells are more primitive than adult-derived stem cells (Hass et al., 2011; Kwon et al., 2016). Previously, we compared the expression profiles of umbilical cord-derived MSCs (UC-MSCs), fetal placenta-derived MSCs (FP-MSCs), and umbilical cord placental junction-derived MSCs (CPJ-MSCs) from three different parts of perinatal tissue. The results showed that transcriptomic differences exist among the three types of MSCs derived from perinatal tissues, the genes related to inflammation and immune regulation in UC-MSCs were very differentially expressed compared to those in CPJ-MSCs and FP-MSCs, and the genes related to cell cycle were highly expressed in FP-MSCs but were low expressed in UC-MSCs (Li et al., 2022). Amidst over a thousand MSC-involved FDA-registered clinical trials focusing on tissue repair, regeneration, immunomodulation, and graft-versus-host disease (Levy et al., 2020), no conclusive evidence delineates the ideal MSC type for OA treatment.

Preceding research has showcased MSCs’ immunoregulatory prowess under inflammatory stimuli (Lee et al., 2010), with priming enhancing immune potential. Inflammatory factor priming steers cells toward an anti-injury phenotype, optimizing OA treatment response. Cartilage regeneration is heavily influenced by inflammatory cytokines, fostering MSC chondrogenic differentiation (Yang et al., 2018). Yet, a comparative assessment of IL-1β priming efficacy across different MSC types in OA therapy remains absent.

Our study isolates MSCs from three distinct perinatal tissue types: UC-MSCs, FP-MSCs and CPJ-MSCs. IL-1β-primed MSCs are co-cultured with OA-like chondrocytes, their effect on recovery properties examined. This research aids in discerning optimal cell sources for OA treatment and provides an IL-1β priming reference for future preclinical and clinical investigations.

## 2 Materials and methods

### 2.1 MSCs isolation and culture

The protocol employed in this study was endorsed by the ethics committee of The First People’s Hospital of Yunnan Province (Approval Code: KHLL2018-GXB002), and written informed consent was obtained from the donors before tissue donation. Human perinatal tissue samples (*n* = 3) were procured from fetuses via cesarean section at The First People’s Hospital of Yunnan Province. Three distinct anatomical regions of perinatal tissue, namely, the umbilical cord, fetal placenta, and umbilical cord placental junction, were manually dissected. Utilizing the direct explant method, UC-MSCs, FP-MSCs, and CPJ-MSCs were isolated, and the explants were subsequently subjected to culture for MSC derivation (Li et al., 2022). All the three kinds of MSCs were cultured with the DMEM medium (Gibco, United States) supplemented with 12% fetal bovine serum (Gibco, United States) and 1% penicillin/streptomycin (Gibco, United States).

### 2.2 Flow cytometric analysis for surface marker profiles of MSCs

To discern the surface markers of the three types of MSCs (UC-MSCs, FP-MSCs, and CPJ-MSCs), the following protocol was employed: Approximately 1×10^6^ cells were introduced into individual flow tubes. The cells underwent two rounds of cold phosphate-buffered saline (PBS) washing. Subsequently, an MSC identification Kit (BD Bioscience) was employed for the characterization of MSCs. The cells were incubated with specific antibodies, including CD44, CD90, CD73, CD105, and a negative cocktail, for a duration of 30 min on ice in a dark environment as per the manufacturer’s instructions. The resulting cells were analyzed utilizing a flow cytometry instrument, specifically the FACS Calibur system from BD Bioscience, United States. The acquired data were subjected to analysis using the FlowJo software (Version 10).

### 2.3 Evaluation of the differentiation capacity of MSCs

Following the provided guidelines, commercial differentiation kits sourced from R&D Systems, United States, were employed to assess the differentiation potential of MSCs derived from human perinatal tissue into adipocytes, osteoblasts, and chondrocytes.

Adipogenic and Osteogenic Differentiation: MSCs were seeded in a 6-well plate at a density of 1×10^6^ cells per well. Adipogenic and osteogenic differentiation media were replaced every 48 h over a 21-day period. Subsequently, the cells were fixed with paraformaldehyde (PFA) from Biosharp, China, and subjected to staining. Oil Red O and Alizarin red solutions were employed to visualize adipocyte and osteoblast differentiation, respectively.

Chondrogenic Differentiation: 2.5×10^5^ MSCs were placed in a 15 mL centrifuge tube. Centrifugation was performed at 1,000 rpm for 3 min to form a compact cell pellet. Subsequently, the cell pellet were cultured in chondrogenic differentiation medium for a duration of 21 days, with the medium refreshed bi-daily. Following the differentiation period, cell deposition sections embedded in OCT (Sakura, Japan) were stained using Alcian blue to detect chondrogenic differentiation.

### 2.4 Conditioned medium formulation

A control culture medium was prepared using DMEM (Gibco, United States) supplemented with 12% FBS (Gibco, United States). To create an inflammatory environment, pro-inflammatory cytokines IL-1β (R&D Systems, United States) were introduced into the control medium, resulting in a conditioned culture medium. The final concentration of IL-1β in the conditioned medium was adjusted to 20 ng/mL (Yang et al., 2018).

### 2.5 Proliferation assay

Ki67 immunofluorescence staining was employed to assess the proliferation capacity of the three MSCs following IL-1β stimulation (Utami et al., 2020; Li et al., 2021). Cells were fixed using 4% paraformaldehyde (PFA) at room temperature for 20 min, followed by permeabilization using 0.1% Triton X-100 (Sigma, United States) for 5 min. Subsequently, cells were incubated overnight at 4°C with the Ki67 antibody (Abcam, United Kingdom). Following a cold PBS wash, the cells were treated with an HRP-labeled secondary antibody and 4,6-diamidino-2-phenylindole (DAPI) for 2 h. Finally, observation was conducted using a laser scanning confocal microscope (Leica Microsystems, United States) at an appropriate magnification.

### 2.6 Cell migration assay

Place each of the three MSC types in separate wells of a six-well plate, using both the control cultivation medium and the conditioned medium. After incubating for 24 h, the MSCs reached near confluence. Subsequently, a fixed region of cells was gently scratched using a pipette tip (Axygen, United States). Following this, the cells were subjected to two washes with PBS and then treated with 10 μg/mL mitomycin C (Sigma, United States) for a duration of 3–4 h (Diem et al., 2020), After the mitomycin C treatment, the cells were replenished with appropriate fresh medium. The migration of cells within the scratched area was observed and recorded at the 24-hour mark using a microscope (Leica Optical, United States).

### 2.7 RNA isolation and RT-qPCR

The total RNA of the cells with different treatments were extracted by using TRIzol reagent (Invitrogen, United States) according to the manufacturer’s instructions. Read the concentration of total RNA by a microplate reader, and transcribe about 1ug of RNA into cDNA using a RT reagent Kit with gDNA Eraser (Takara, Japan) according to the manufacturer’s protocol. RT-qPCR was performed with SYBR Green Master Mix (Thermo Fisher Scientific, United States). Glyceraldehyde-3-phosphate dehydrogenase (GAPDH) was used as an internal reference gene and the relative expression of mRNA was calculated by 2 ^−ΔΔCt^ method. The sequences of all PCR primers were listed in Supplementary Table S1.

### 2.8 Primary culture of ACs and *in vitro* model of OA-like ACs

Rat ACs were isolated from the knee joints of male Sprague-Dawley rats weighting 250 ± 20 g (n = 20, provided by the Experimental Animal Center of Kunming Medical University), and all animal works were approved by the animal ethics committee of State Key Laboratory of Primate Biomedical Research (LPBR202004005). The ACs were isolated by 0.25% trypsin (Gibco, United States) and type Ⅱ collagenase (sigma, United States), cultured with DMEM/F12 medium (Gibco, United States) containing 10% FBS, and were identified by Toluidine Blue (Solarbio, China) and COLⅡ (abcam, United Kingdom) immunofluorescence staining. The second generation of ACs were used in all experiments.

For the *in vitro* model of OA-like ACs, H_2_O_2_ (Jing Rui, China) was added to the ACs medium to induce ACs expressing an OA-like phenotype (ACs + H_2_O_2_) (Ma et al., 2022; Chen et al., 2023; Yagi et al., 2023). Briefly, the ACs were treated with the conditioned medium for 24 h. Then, the OA-like ACs were co-cultured with different MSCs before and after IL-1β treatment (UC-MSCs, UC-MSCs + IL-1β, FP-MSCs, FP-MSCs + IL-1β, CPJ-MSCs, CPJ-MSCs + IL-1β). ACs without treatment served as blank controls. After co-culturing for 48 h (Zhu et al., 2017; Zhang et al., 2018), the changes of biological properties of ACs in each group (ACs, ACs + H_2_O_2_, UC-MSCs + ACs + H_2_O_2_, UC-MSCs + IL-1β+ACs + H_2_O_2_, FP-MSCs + ACs + H_2_O_2_, FP-MSCs + IL-1β+ACs + H_2_O_2_, CPJ-MSCs + ACs + H_2_O_2_ and CPJ-MSCs + IL-1β+ACs + H_2_O_2_) were assessed.

### 2.9 Western blot

Total protein was isolated from the ACs with different treatments using RIPA protein lysis buffer (Solarbio, China), and protein concentrations were determined by a BCA assay kit (Beyotime, China). Briefly, the protein samples were separated by sodium dodecyl sulfate-polyacrylamide gel electrophoresis (SDS-PAGE) under denaturing conditions and then transferred to PVDF membranes (Millipore, United States). After blocking with 5% skimmed milk at room temperature for 2 h, the membranes were incubated with anti-matrix metallopeptidase 13 (MMP13) antibody (abcam, United Kingdom), anti-MMP3 antibody (abcam, United Kingdom), anti-SRY-box9 (SOX9) antibody (abcam, United Kingdom) and anti-GAPDH antibody (abcam, United Kingdom) overnight at 4°C. After being washed with Tris-buffered saline and tween 20 (TBST, Yeasen, Shanghai, China) for 3 times, the membranes were then incubated with the secondary antibody (goat anti-rabbit IgG-HRP, abcam, United Kingdom) for 2 h under room temperature. The protein bands were exposed using ChemiDoc MP (Bio-Rad, United States). The gray value of protein bands was determined using ImageJ (National Institutes of Health, United States).

### 2.10 Statistical analysis

Data are expressed as the mean ± SEM. Statistical significances were analyzed by GraphPad Prism 8 (GraphPad Software, United States), using Student’s t-test for the comparison of two groups. Statistical significance was set at *p* < 0.05.

## 3 Results

### 3.1 Isolation and identification of UC-MSCs, FP-MSCs, and CPJ-MSCs

**FIGURE 1:**
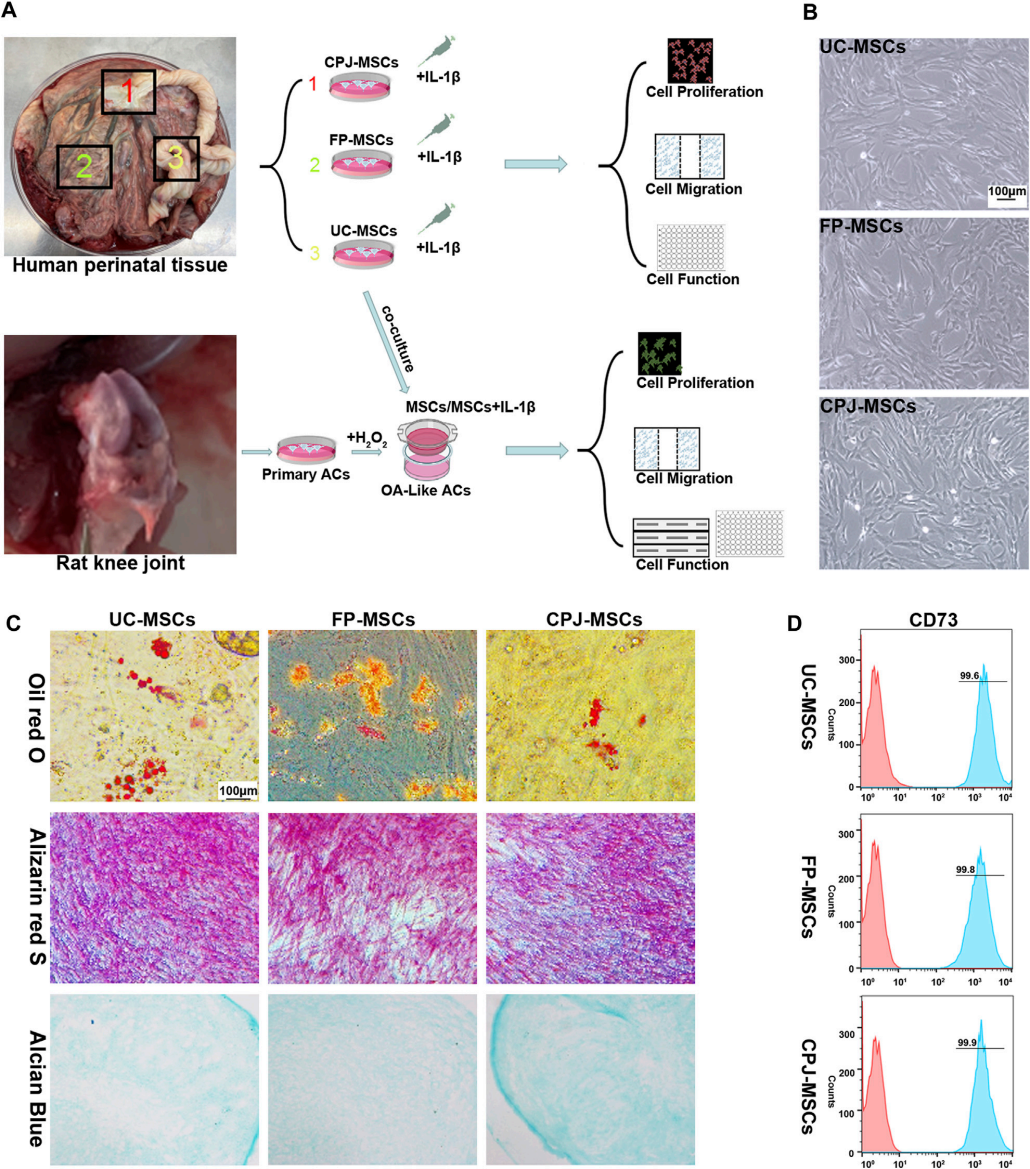
Isolation and identification of UC-MSCs, FP-MSCs and CPJ-MSCs (*n* = 3). **(A)** The experimental procedure of this study. **(B)** All MSCs exhibited a similar cell morphology. Scale bar: 100 μm. **(C)** All the three types of MSCs were positive for oil red O (adipogenic differentiation), alizarin red S (osteogenic differentiation) and alcian blue (chondrogenic differentiation). Scale bar: 100 μm. **(D)** Flow cytometric analysis of CD73 expression in different MSCs.

The experimental procedure of this study was shown in Figure 1A. According to the International Society for Cellular Therapy (ISCT) criteria (Dominici et al., 2006), the MSCs derived from different parts of human perinatal tissue (UC-MSCs, FP-MSCs and CPJ-MSCs) exhibited typical fibroblastoid and spindle-shaped morphology (Figure 1B). Using flow cytometry to examine the classical MSCs surface markers defined by the ISCT criteria (including CD44, CD73 and CD90), which the positive expression rates were all above 95% (Figure 1D; Supplementary Figure S1). The cells also presented the ability to differentiate into adipogenic, osteogenic and chondrogenic (Figure 1C).

### 3.2 IL-1β priming enhance the biological properties of MSCs

**FIGURE 2:**
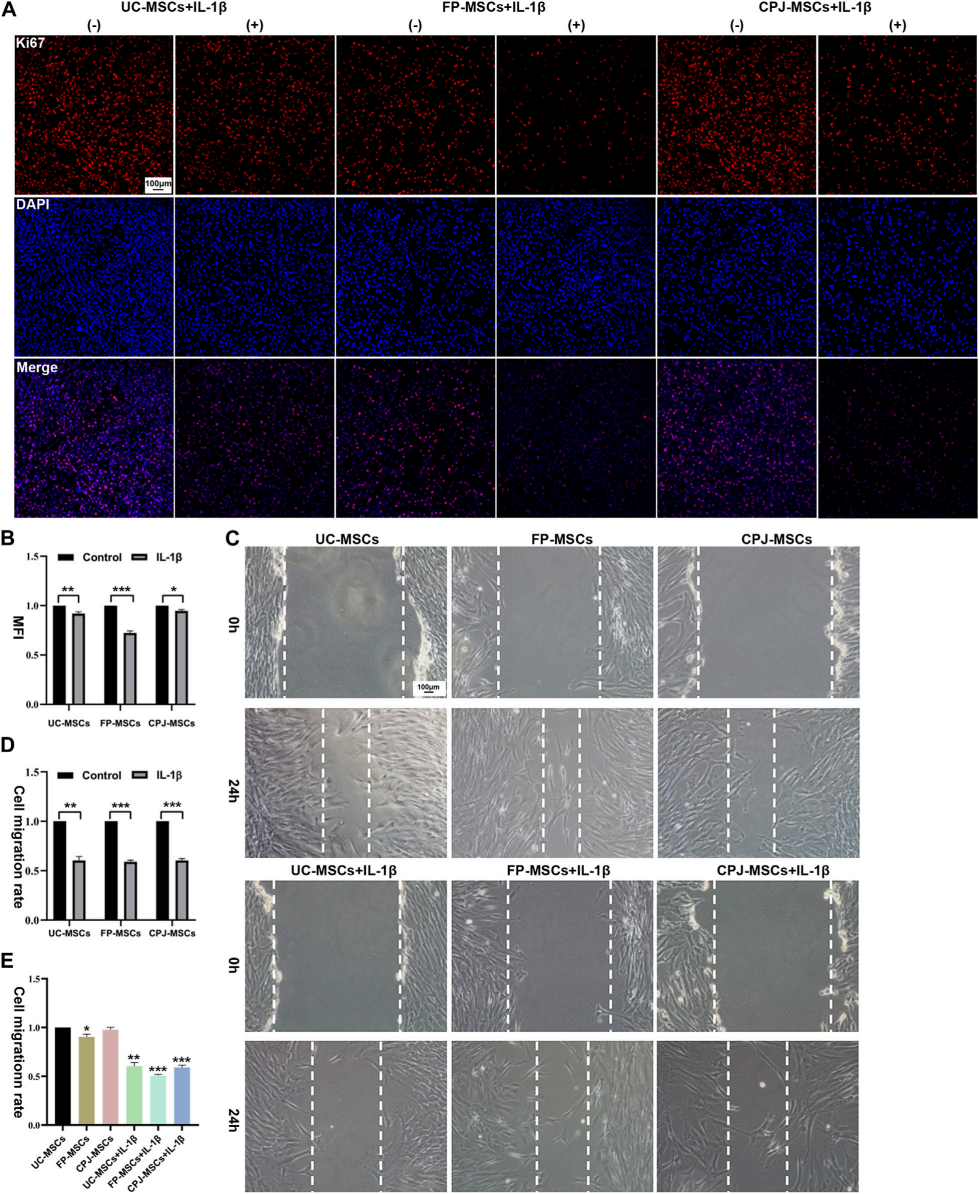
IL-1β can alter the biological properties of MSCs (*n* = 3). **(A)** Immunofluorescence staining of MSCs (Ki67) showed the effects of IL-1β on UC-MSCs, FP-MSCs and CPJ-MSCs proliferation. (−) stands for MSCs without the treatment of IL-1β and (+) means the MSCs were treated with IL-1β. Scale bar: 100 μm. **(B)** The mean fluorescence intensity (MFI) of MSCs (Ki67) were counted with ImageJ and the experiment was repeated three times and values represent means ± SEM of three donors. *p < 0.05, **p < 0.01, ***p < 0.001 vs. control group. **(C)** Scratch tests were used to show the effects of IL-1β on UC-MSCs, FP-MSCs and CPJ-MSCs migration. Scale bar: 100 μm. **(D)** The cell migration rate of MSCs were counted with ImageJ and the experiment was repeated three times and values represent means ± SEM of three donors. **p < 0.01, ***p < 0.001 vs. control group. **(E)** Counting the cell migration rate again with ImageJ. *p < 0.05, **p < 0.01, ***p < 0.001 vs. UC-MSCs group.

Using Ki67 immunofluorescence staining to evaluate the changes of MSCs proliferation *in vitro*, after UC-MSCs, FP-MSCs and CPJ-MSCs were treated with IL-1β for 24 h (Figure 2A). ImageJ was used to count the mean fluorescence intensity (MFI) of each group of MSCs. The results showed that the inflammatory environment of IL-1β could inhibit the proliferation of MSCs (*p* < 0.05). Among them, pretreatment of IL-1β had the greatest effect on the proliferation capacity of FP-MSCs (Figure 2B).

Scratch assay was used to evaluate the changes of migration capability of three kinds of MSCs before and after IL-1β treatment (Figure 2C). The results revealed that after stimulation of the IL-1β-induced inflammatory environment within 24 h impaired the migration ability of MSCs compared to the control group (*p* < 0.01). More precisely, the migration ability of FP-MSCs is more affected by IL-1β than UC-MSCs and CPJ-MSCs (Figure 2D). We also found that the cell migration rate of FP-MSCs was slower than that of the other two strains (*p* < 0.05) (Figure 2E).

We also measure the mRNA expression of cytokines in three types of MSCs after they were treated with IL-1β, including chemokine (C-C motif) ligand2 (CCL2), IL6, Programmed cell death ligand1(PDL1), insulin-like growth factor2 (IGF2), bone morphogenetic protein 2 (BMP2) and SRY-box9 (SOX9) (Supplementary Figure S2). We found that the expression of CCL2, IL6, PDL1 and IGF2 were all increased in IL-1β group, which suggested that IL-1β-induced inflammatory environment may enhance the immunomodulatory capacity of MSCs. We also found that IL-1β can upregulate the expressions of SOX9 and BMP2 in MSCs, which suggests that MSCs treated with IL-1β may have a better therapeutic effect on OA. However, only some of them showed significant differences in gene expression after IL-1β stimulation, which may be due to the small number of samples in this experiment or the large individual differences between different donors.

### 3.3 Extraction and characterization of ACs and *in vitro* model of OA-like ACs

**FIGURE 3:**
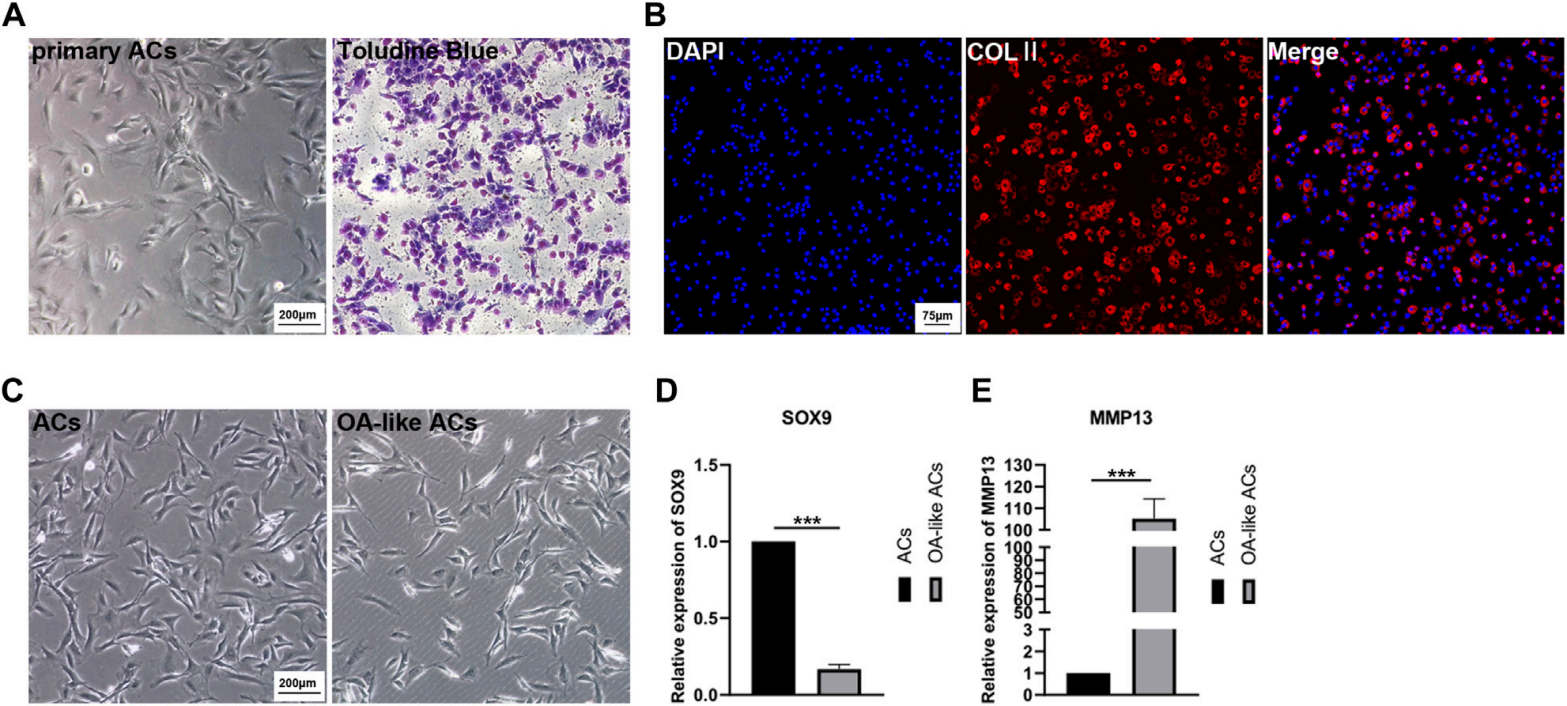
Primary culture of ACs and *in vitro* model of OA-like ACs. **(A)** Primary ACs isolated from rat knee joints cartilage, and then identified ACs inpassage 2 with Toluidine Blue. Scale Bar: 200 μm. **(B)** Identification of ACs using immunofluorescence staining of COLⅡ. Scale Bar: 75 μm. **(C)** Induced ACs with H_2_O_2_ to establish an OA-like ACs model *in vitro*. Scale Bar: 200 μm. **(D,E)** The expression level of SOX9 and MMP13 after ACs were induced by H_2_O_2_. ***p < 0.001 vs. ACs group (*n* = 3).

The ACs isolated from cartilage tissue were cultured in cell culture dishes, then passaging the cells after the they reached 80% confluence while initially identifying them with Toluidine Blue (Figure 3A). Collagen Ⅱ (COLⅡ) immunofluorescence staining was determined in order to further identify the ACs (Figure 3B).

After ACs were treated with 3% H_2_O_2_ for 24 h, we can clearly observe a decrease in the number of cells (Figure 3C), and the expression of SOX9 and matrix metallo proteinase 13 (MMP13) were determined to assess the OA-like ACs model (Figures 3D, E). These results implied that inflammatory ACs were successfully established by treatment with H_2_O_2_.

### 3.4 The effects of MSCs from three sources before and after IL-1β treatment on OA-like ACs

**FIGURE 4:**
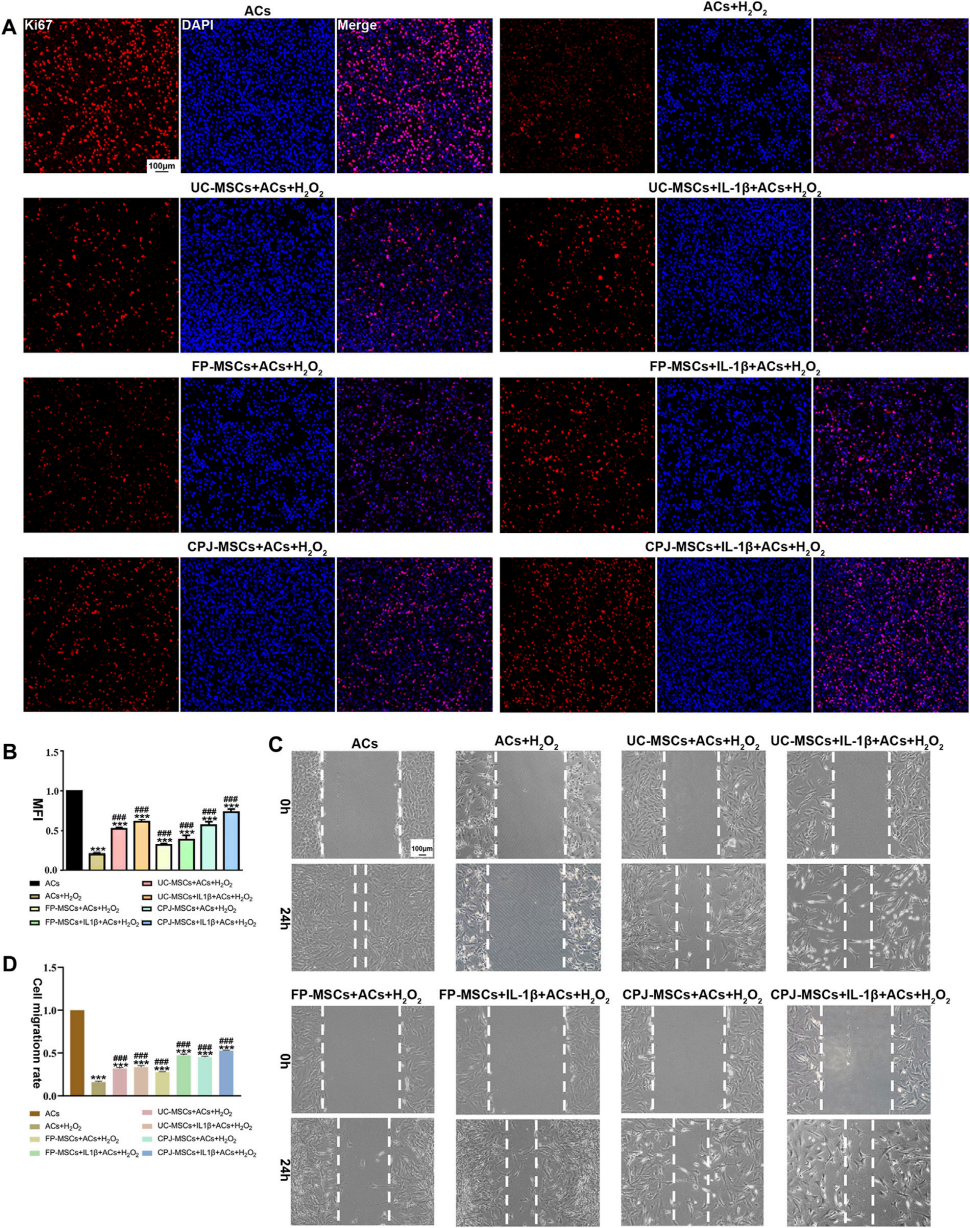
The proliferative and migratory abilities of OA-like ACs were somewhat restored after co-cultured with different MSCs (*n* = 3). **(A)** Immunofluorescence staining of ACs (Ki67) showed the effect of different MSCs on injured ACs proliferation. Scale bar: 100 μm. **(B)** The mean fluorescence intensity (MFI) of Acs (Ki67) were counted with ImageJ. ***p < 0.001 vs. ACs group; ^###^ *p* < 0.001 vs. ACs + H_2_O_2_ group. **(C)** Using scratch tests to show the effects of MSCs on injured ACs migration. Scale bar: 100 μm. **(D)** The cell migration rate of ACs were counted with ImageJ. ***p < 0.001 vs. ACs group; ^###^ *p* < 0.001 vs. ACs + H_2_O_2_ group.

The OA-like ACs were co-cultured with three different MSCs before and after IL-1β treatment for 48 h. To understand the effects of MSCs in OA-like ACs, the proliferation ability and migration ability of cells were determined. By analyzing the MFI of each group of cells, we found that H_2_O_2_ treatment significantly inhibited the proliferation ability of ACs (*p* < 0.05). When the OA-like ACs were co-cultured with different MSCs, the proliferation ability of the ACs was significantly increased compared with the H_2_O_2_ group (*p* < 0.05), and when the OA-like ACs co-cultured with IL-1β+CPJ-MSCs, the proliferation ability of the ACs was higher than other groups (Figures 4A, B). For cell migration, the H_2_O_2_ group was markedly lower than that in the blank control group (*p* < 0.05), while after treatment with MSCs the cell migration rate was restored to a similar level to that of the blank control group, and among them, the IL-1β+CPJ-MSCs has the best effect on restoration of OA-like ACs migration ability (Figures 4C, D). Taken together, H_2_O_2_ treatment suppressed the proliferation ability and the migration ability of ACs, whereas MSCs could properly restore the ability of cell proliferation and migration induced by H_2_O_2_, and among them, IL-1β+CPJ-MSCs shows the best restoration capabilities.

**FIGURE 5:**
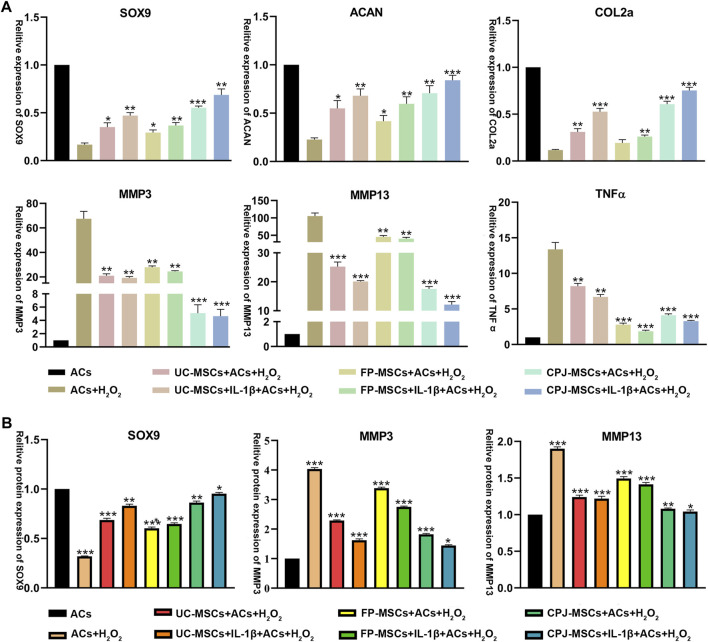
The expressions of SOX9, ACAN, COLⅡ, MMP3, MMP13, and TNFα in OA-like ACs co-cultured with different MSCs (*n* = 3). **(A)** The level of related gene expression in OA-like ACs co-cultured with different MSCs including SOX9, ACAN, COLⅡ, MMP3, MMP13, and TNFα. *p < 0.05, **p < 0.01, ***p < 0.001 vs. ACs + H_2_O_2_ group. **(B)** The level of related protein expression in OA-like ACs co-cultured with different MSCs including SOX9, MMP3 and MMP13. *p < 0.05, **p < 0.01, ***p < 0.001 vs. ACs group.

To further investigate the molecular mechanisms by which different MSCs affect ACs, the expression of SOX9, ACAN, COLⅡ, MMP3, MMP13 and TNFα was examined by RT-qPCR. The results showed that all MSCs could repair OA-like ACs. MSCs can significantly upregulated the expression of cartilage synthesis-related factors SOX9, ACAN and COLⅡ, downregulated the expression of cartilage catabolic markers MMP3 and MMP13, and also reduce the expression of inflammatory factor TNFα. All the improvement effect is significantly different (*p* < 0.05). Among them, from the perspective of balancing metabolic activity of cartilage and the chondrogenic differentiation, IL-1β+CPJ-MSCs has the most obvious repair effect on ACs, and IL-1β+FP-MSCs can reduce the expression of inflammatory factors better result (Figure 5A). At the same time, we also extracted proteins from different groups of cells and performed Western blot to verify again some of the cytokines which has already detected in RT-qPCR, the results also showed that IL-1β+CPJ-MSCs had the best repairing effect on ACs (Figure 5B; Supplementary Figure S3).

## 4 Discussion

Articular cartilage defects in osteoarthritis have emerged as an intricate medical challenge, with limited available therapeutic strategies. In this study, we embarked on isolating UC-MSCs, FP-MSCs, and CPJ-MSCs from distinct perinatal tissue sections. We investigated the influence of an IL-1β-induced inflammatory milieu on MSCs and the subsequent regulatory effects of diverse MSCs on OA-like ACs. Our *in vitro* investigations illuminated that the restorative impact of MSCs on ACs was heightened upon IL-1β pre-treatment. This suggests that IL-1β priming can enhance the therapeutic efficacy of MSCs in clinical applications.

Furthermore, the study divulged dissimilarities among the three MSC types derived from identical perinatal tissues concerning their efficacy in repairing OA-like ACs. Under IL-1β exposure, CPJ-MSCs demonstrated a greater capacity to stimulate AC proliferation and migration, bolster cartilage anabolism, and mitigate cartilage degradation. Conversely, IL-1β-treated FP-MSCs exhibited a superior ability to alleviate inflammation. These observations point towards CPJ-MSCs and FP-MSCs as being optimal cell sources for OA treatment.

While various MSC sources have been applied in OA therapy, their individual characteristics are worth considering. Although certain similarities exist among MSCs from diverse tissue sources (Dominici et al., 2006), distinct properties also exist. Presently, MSCs from different origins display divergent proliferation and differentiation capacities (Beeravolu et al., 2017), along with variations in paracrine effects (Wu et al., 2018). This hints at the possibility of divergent clinical indications resulting from MSC heterogeneity. Studies have demonstrated, for instance, that infrapatellar fat pad-derived MSCs (IPFP-MSCs) exhibit superior chondrogenic potential *in vitro* and *in vivo* when compared to bone marrow-derived mesenchymal stem cells (BMSCs) (Liao et al., 2022). Similarly, placenta basal decidua-derived MSCs (DC-MSCs) have shown heightened immunomodulatory capabilities in comparison to UC-MSCs (Guan et al., 2019). Interestingly, chorionic plate-derived MSCs (CP-MSCs) display greater expression of adipogenesis-related genes when compared to Wharton’s jelly-derived MSCs (WJ-MSCs), and the latter express more mature liver cell markers than CP-MSCs (Kim et al., 2011). Additionally, amniotic membrane-derived MSCs (AM-MSCs) exhibit heightened osteogenic differentiation efficiency under serum-free conditions, while CP-MSCs excel in adipogenic differentiation and proliferation (Ma et al., 2019). Despite these findings, there remains an absence of research pinpointing the most suitable perinatal tissue-derived MSC source for OA treatment.

Besides their remarkable differentiation potential, MSCs also possess potent inflammation-regulatory attributes (Gazdic et al., 2015; Vasanthan et al., 2020). However, these anti-inflammatory qualities are often a product of inflammatory cytokine influence rather than an inherent trait of MSCs themselves (Ren et al., 2008). MSCs can secrete an array of immune modulators and cell growth factors within an inflammatory environment, promoting stem cell-driven tissue repair within damaged regions (Prockop and Oh, 2012). For instance, interferon-γ (IFN-γ) can upregulate hepatocyte growth factor (HGF) and transforming growth factor-β1 (TGFβ1) expression in MSCs, while also inducing indoleamine (2,3)-dioxygenase (IDO) production, which enhances MSC immune regulation (Ryan et al., 2007). This is substantiated by studies showing that IFN-γ and poly (I:C) pre-treatment can enhance MSCs therapeutic efficacy in dextran sodium sulfate-induced colitis in mice (Lim et al., 2021). While, TNF-α can increases the expression of various proteases and protease inhibitors in MSCs, including cysteine protease cathepsin isoforms and MMPs, and also increases the expression of IL-6, IL-8, monocyte chemotactic protein-1 (MCP-1) and chemokine (C-X-C motif)ligand 6 (CXCL6) (Lee et al., 2010). Despite these insights, no studies have investigated the comparative efficacy of IL-1β priming across different MSC sources in OA treatment.

Previous research has indicated that IL-1β can simultaneously suppress osteogenic mineralization while augmenting cartilage differentiation in human MSCs (Yang et al., 2018; Mo et al., 2022). Moreover, the joint cavity of OA patients is often characterized by an inflammatory environment mediated by interleukins like IL-1β (Chow and Chin, 2020). Therefore, we use IL-1β activate three different MSCs and evaluate their effects on OA like cells *in vitro*, further demonstrating the heterogeneity of MSCs from different tissue sources. Our results showcased that IL-1β-induced inflammation diminished the proliferative and migratory capabilities of all three MSC types, while concurrently upregulating the expression of CCL2, IL6, PDL1, and IGF2-entities capable of mitigating ongoing inflammation (Petri et al., 2017; Harrell et al., 2019). The inflammatory milieu also spurred increased SOX9 and BMP2 expressions in MSCs, fostering chondrogenic differentiation while bolstering regulatory abilities. Our findings suggest that IL-1β-treated MSCs hold potential advantages for OA treatment.

However, this study presents certain limitations. We omitted the establishment of an OA animal model to validate the therapeutic efficacy of the three MSC types *in vivo*. Additionally, the chemotactic immunity aspects could not be addressed in our *in vitro* experiments. Another limitation involves the decrease in inflammation regulation, proliferation, and differentiation potential of MSCs with age (Zha et al., 2021). Although age screening of donors was omitted in our experiment, it remains a relevant consideration. Individual variability among MSCs, even when isolated from the same tissue source, was also noted (Sun et al., 2020). While the expression trends of MSC cytokines aligned across each group after IL-1β treatment, statistical differences were not observed due to substantial intergroup disparities. To address this, a larger number of human perinatal tissue samples should be included for more comprehensive experiments.

In essence, this study cultivated an *in vitro* cartilage injury environment, contributing to the preliminary selection of the most suitable MSC source for OA treatment. These insights pave the way for future *in vivo* experimentation using animal models and offer a reference point for forthcoming clinical research endeavors.

## 5 Conclusion

MSC-based therapies offer a promising avenue for addressing various degenerative disorders due to their safety profile, immunosuppressive capabilities, and capacity to sense and home in on inflamed regions. This unique combination holds the potential to significantly enhance cartilage recovery and repair in cases of osteoarthritis (OA). In our investigation, we unveil the transformative impact of preconditioning on three distinct MSC types. While preconditioning diminishes the proliferation and migration of these MSCs, it concurrently amplifies their immunomodulatory and chondrogenic potential, fostering cartilage repair. This transition towards an anti-inflammatory and pro-regenerative phenotype is of paramount significance. Moreover, our research unveils the most adept MSC types in reducing inflammatory factor expression and enhancing the health of OA-like ACs. By systematically identifying the optimal cell sources and pre-treatment strategies, our findings hold considerable value in advancing OA therapy, offering a robust foundation for subsequent preclinical investigations.

## Data Availability

The original contributions presented in the study are included in the article/Supplementary Material, further inquiries can be directed to the corresponding authors.
