# Supplementary material for: Augmenting mesenchymal stem cell therapy for osteoarthritis via inflammatory priming: a comparative study on mesenchymal stem cells derived from various perinatal tissue sources
Source: Front Cell Dev Biol. 2023 Oct 4;11:1279574. doi: 10.3389/fcell.2023.1279574 (PMC10582349; doi:10.3389/fcell.2023.1279574)
Supplement: Supplementary file 1 [file Table1.DOC]

Supplementary Material

### Supplementary Table S1 The sequences of all primers used for RT-qPCR.

| species | primers |  | sequences(5′–3′) |
| --- | --- | --- | --- |
| human | GAPDH | Forward | CAACGTGTCAGTGGTGGACCTG |
|  |  | Reverse | GTGTCGCTGTTGAAGTCAGAGGAG |
| human | SOX9 | Forward | TCCCCGCAACAGATCTCCTA |
|  |  | Reverse | AGCTGTGTGTAGACGGGTTG |
| human | BMP2 | Forward | CGTCAAGCCAAACACAAAC |
|  |  | Reverse | CAATCCAGTCATTCCACCC |
| human | CCL2 | Forward | AGAATCACCAGCAGCAAGTGTCC |
|  |  | Reverse | TTGCTTGTCCAGGTGGTCCATG |
| human | IL6 | Forward | ACAGCCACTCACCTCTTCA |
|  |  | Reverse | ATGTCTCCTTTCTCAGGGC |
| human | PDL1 | Forward | ACTGGCATTTGCTGAACG |
|  |  | Reverse | GATGGTCACTGCTTGTCCA |
| human | IGF2 | Forward | CATCGTTGAGGAGTGCTGT |
|  |  | Reverse | TTGGAAGAACTTGCCCAC |
| rat | GAPDH | Forward | GCTGCCTTCTCTTGTGACA |
|  |  | Reverse | TTGAACTTGCCGTGGGTA |
| rat | SOX9 | Forward | GGAGGAAGTCGGTGAAGAAT |
|  |  | Reverse | TGAAGATGGCGTTAGGAGAG |
| rat | ACAN | Forward | TCTTCGCCACACAAATGG |
|  |  | Reverse | ACCAGCCAGCATAGCACTT |
| rat | COLⅡ | Forward | GCAAGGAGAAGAAGCACATC |
|  |  | Reverse | ACAGTAGACGGAGGAAAGTCA |
| rat | MMP3 | Forward | CCTTGGGCTGAAGATGACA |
|  |  | Reverse | TCCATTTGGGTGAACCTGG |
| rat | MMP13 | Forward | TTTGGCTTAGATGTGACTGG |
|  |  | Reverse | TGGGACCATTTGAGTGTTC |
| rat | TNFα | Forward | GAGATGTGGAACTGGCAGA |
|  |  | Reverse | ATGAGAAGAGGCTGAGGCA |

**
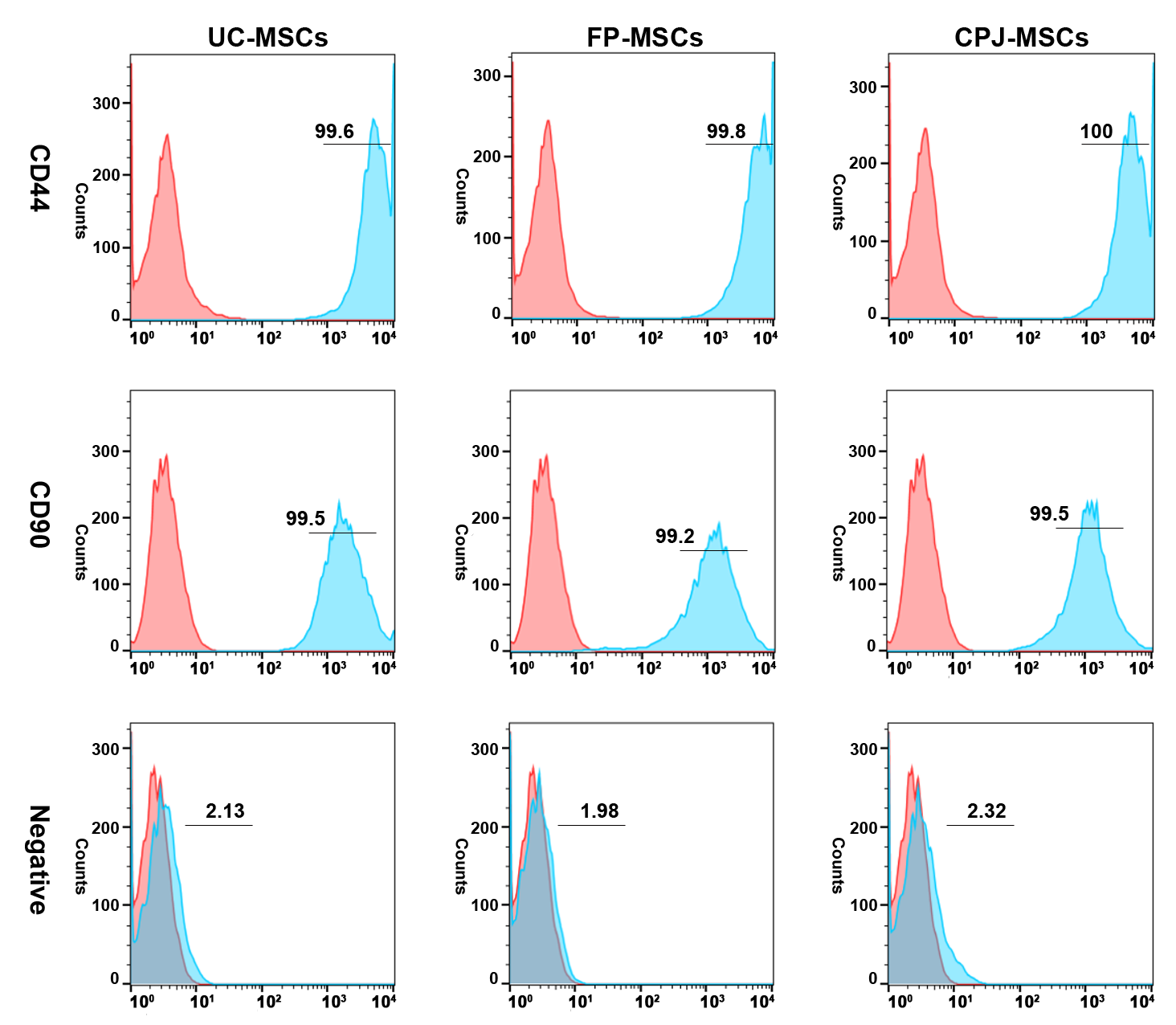
**

**Supplementary FigureS1.** Flow cytometric analysis of CD44 and CD90 expression in different MSCs.


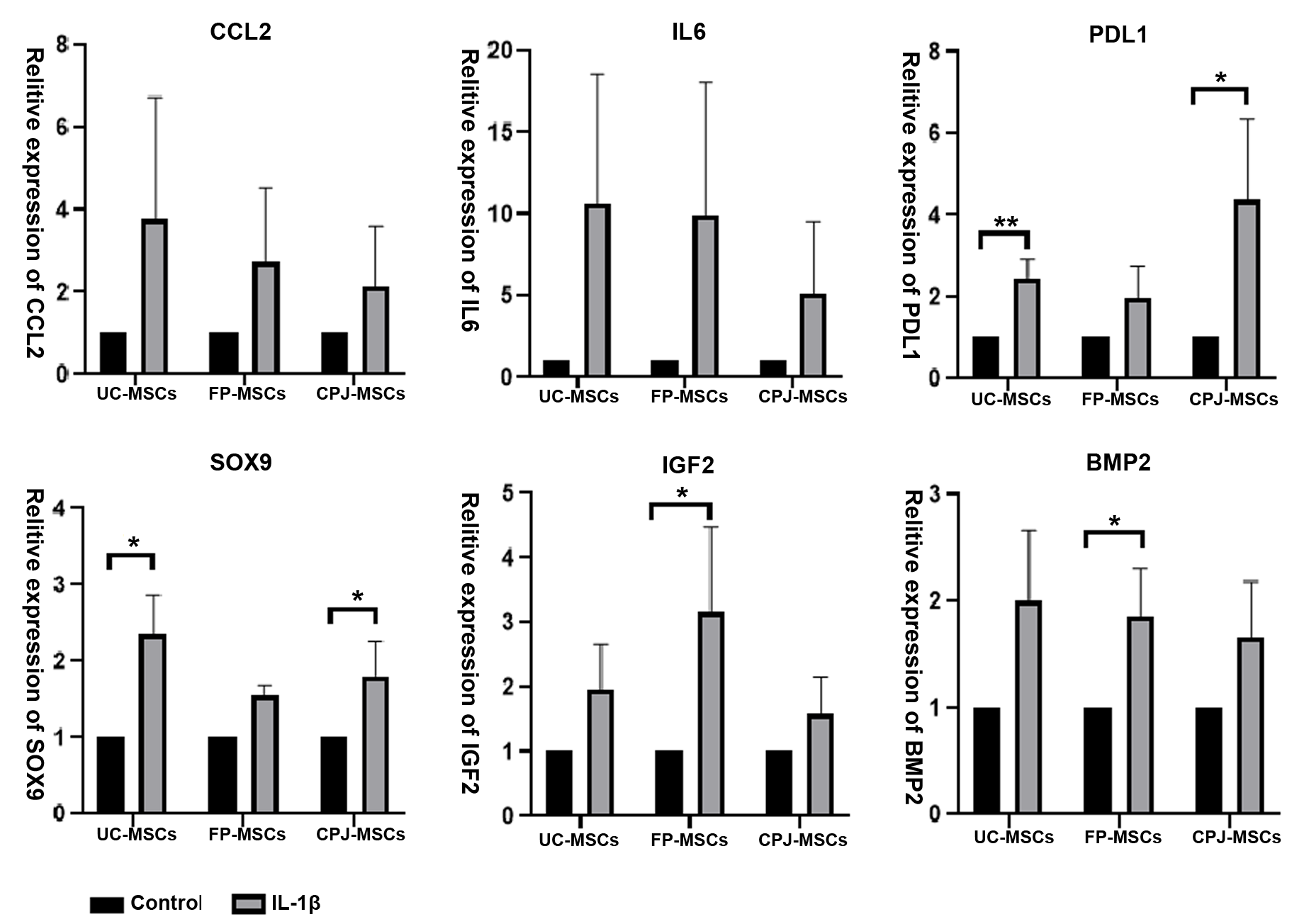


**Supplementary Figure S2.** The expression level of CCL2, IL6, PDL1, SOX9, IGF2 and BMP2 after MSCs were induced by IL-1β. **P* < 0.05, ***P* < 0.05 vs. control group.(n=3).


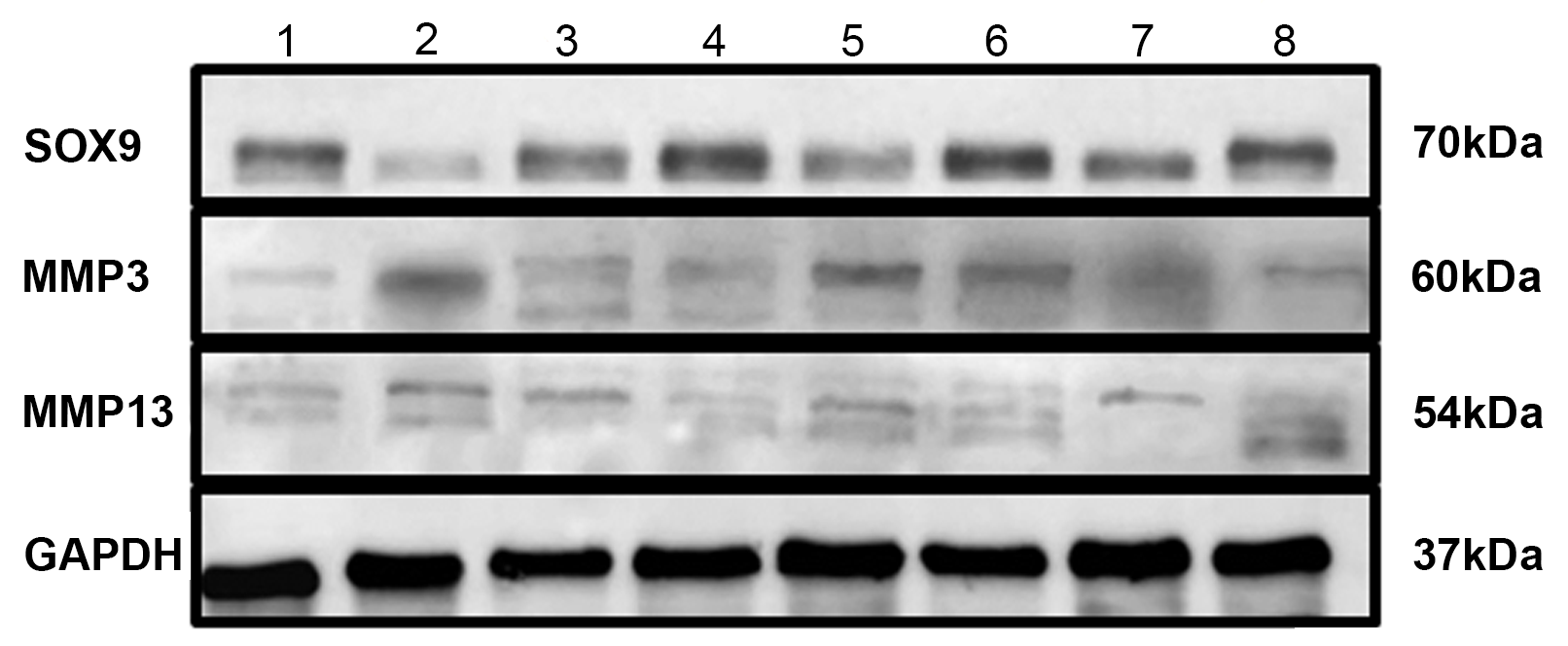


**Supplementary Figure S3.** The protein bands acquired by western blot are ACs, ACs+H_2_O_2_, UC-MSCs+ACs+H_2_O_2_, UC-MSCs+IL-1β+ACs+H_2_O_2_, FP-MSCs+ACs+H_2_O_2_, FP-MSCs+IL-1β+ACs+H_2_O_2_, CPJ-MSCs+ACs+H_2_O_2_ and CPJ-MSCs+IL-1β+ ACs+H_2_O_2_ in order from1-8.
